# Book Reviews

**Published:** 1900-12

**Authors:** 


A TEXT-BOOK OF PRACTICAL MEDICINE By WILLIAM GILMAN THOMPSON, M. D., Professor of Medicine in Cornell University Medical College, New York City; Physician to the Presbyterian and Bellevue Hospitals, New York. In one magnificent 8vo volume of 1010 pages, with 79 engravings. Lea Brothers & Co., Publishers. 1900. [Price, cloth, $5.00; leather, $6.00; half Morocco, $6.50.]

This is one of several excellent contributions to the literature of internal medicine that recently have been made in this country. Professor Thompson is a teacher of experience, who well knows the needs of students as well as practitioners of medicine. It is strictly speaking a modern text-book, presenting all the most recent facts and theories relating to the origin of diseases, their causation, pathology, prevention and treatment. Clinical microscopy and bacteriology occupy a prominence commensurate with their importance.

As might be anticipated in such a work by so experienced a teacher, due prominence is therein given to tuberculosis, a disease that is second to none in importance just now, and which outranks all other infectious diseases in destructiveness. The fact that it is preventable is slowly being recognised by the mass of physicians, and by the more intelligent of the laity. Formerly, this ravaging disease was popularly supposed to limit itself to the lungs, and even the profession has only comparatively recently recognised that it may attack almost any organ or tissue of the body. Thompson has set this latter fact forth most completely and forcefully.

Another section of the work to which we would especially invite attention is that portion which deals with diseases of the gastro-intestinal tract. There has been much advance of late in this class of diseases, which fact has been given due prominence by this author. The newer methods of diagnosis and treatment are duly set forth whereby the general practitioner will derive great aid and comfort in the management of these affections.

Diseases of the blood are attracting more and more attention with advancing time, and more accuracy in diagnosis is demanded. Thompson handles the subject, including diseases of the vascular system, with great skill. Examination of the blood itself becomes so important in very many diseases nowadays that none can afford to neglect it. It is not difficult to train one's self into sufficient expertness in this line to meet the requirements of clinical accuracy.

We might with propriety, if it were necessary, take up each section of the book, and discuss it chapter by chapter, in almost every one of which could be pointed out elements of excellence. This, however, in view of the general knowledge of practical medicine prevalent at the present day, we deem unessential. What the average reader of book notices desires to know is whether the work under consideration is one of scientific value, in which the science and art of medicine is fully maintained up to the present state of our knowledge.

This treatise is precisely one of that kidney, and no mistake can be made by student or practitioner who takes possession of it and adopts it as a guide.

There are numerous fine engravings in it which serve to illustrate with clearness many points under discussion.

---

A Manual of Personal Hygiene. Edited by Walter L. Pyle, A. M., M. D., Assistant Surgeon to Wills Eye and Ear Hospital, Philadelphia; Fellow of the American Academy of Medicine, etc. 337 pages; illustrated. Philadelphia: W. B. Saunders & Co. 1900. [Price, $1.50 net.]

Although this book unpretentiously calls itself a manual, it is really encyclopedic in character, there being seven authors or contributors to as many different sections, each more or less distinct, yet the whole is supervised by the editor, who has succeeded in blending the text into reasonable homogenity.

Dr. Charles G. Stockton, of Buffalo, contributes the first chapter or section, which deals with hygiene of the digestive apparatus. We know of no one more competent to teach or write upon this topic and the distinguished author has acquitted himself with great credit in his presentation of this subject. The contribution is clothed in simple, elegant English, of which the author is a master, and both lay and professional readers will take pleasure in reading what he says.

The hygiene of the skin and its appendages is of great importance. Dr. George Henry Fox, of New York, discusses the subject with acumen. In these days when the advertising "dermatologist" is abroad in the land, it would be well for people of intelligence to think twice before employing one, and meantime read Fox's article.

Dr. E. Fletcher Ingals, of Chicago, a laryngologist of great experience, writes upon hygiene of the vocal and respiratory apparatus; Dr. B. Alex. Randall, of Philadelphia, discourses upon hygiene of the ear; Dr. Walter L. Pyle, of the same city, editor of the book, tells us of the hygiene of the eye, and of the school-room; Dr. J. W. Courtney, of Boston, presents the hygiene of the brain and nervous system, while Dr. G. N. Stewart, of Cleveland, offers a final chapter, which briefly discusses the importance and methods of physical exercise.

This is most happily and emphatically a personal presentation of hygiene in its several bearings and belongs in the library of every well-bred person.

---

THE MEDICAL NEWS VISITING LIST FOR 1901. Weekly (dated, for 30 patients); monthly (undated, for 120 patients per month); perpetual, undated, for 30 patients weekly per year); and perpetual (undated, for 60 patients weekly per year). The first three styles contain 32 pages of data and 160 pages of blanks. The 60-patient perpetual consists of 256 pages of blanks. Each style in one wallet-shaped book, with pocket, pencil and rubber Philadelphia and New York: Lea Brothers & Co. [Price, seal grain leather, $1,25; thumb-letter index, 25 cents extra.]

We speak from experience when we say that this visiting list is one of the most satisfactory books in the market. It is not necessary to discuss the necessity of a visiting list. Every practising physician knows that he cannot conveniently dispense with this useful time-saver. The only question that presents is "which shall I buy?" No mistake will be made and there will be no regret if the *Medical News Visiting List* is selected. The above title gives an excellent general idea of the book, but one unfamiliar with it will be surprised to find how much valuable information of every-day practicality it contains.

DISEASES OF THE CHEST, THROAT AND NASAL CAVITIES. Including physical diagnosis and diseases of the lungs, mediastinum, heart and aorta, laryngology and diseases of the pharynx, larynx, nose, thyroid gland and esophagus. Fourth edition, revised. With 255 illustrations; 8vo, 765 pages. By E. FLETCHER INGALS, A. M., M. D., Professor of Laryngology and Practice of Medicine, Rush Medical College; Professor of Diseases of the Throat and Chest, Northwestern University Woman's Medical School, etc., etc. New York: William Wood & Co. 1900. [Price, muslin, $4.50; sheep, $5.25.]

This standard treatise is again welcome in a new edition. It has always been justly popular with general practitioners, as well as specialists. The author, taking advantage of the demand for new editions, has always kept its material fresh and abreast of the progress of the subjects with which it deals. It is a clear, concise, scientific work, written by a practical, as well as a scientific teacher. Its popularity is destined to continue in this new edition which, among other excellent qualities, has an admirable index,—a matter of no small amount.

---

A TEXT-BOOK OF PRACTICAL THERAPEUTICS. With especial reference to the application of remedial measures to disease and their employment upon a rational basis. By HOBART AMORY HARE, M. D., Professor of Therapeutics and Materia Medica in the Jefferson Medical College of Philadelphia. With special chapters by Drs. G. E. deSchweinitz, Edward Martin and Barton C. Hirst. New (8th) edition. In one 8vo volume of 796 pages, with 37 engravings and 4 colored plates. Philadelphia and New York: Lea Brothers & Co. 1900. [Price, cloth, $4.00; leather, $5.00 net.]

The unprecedented demand for this text-book has been met by putting forth eight editions of it in ten years, —an almost unparalleled record in medical book printing. Each time the author has improved it, bringing it forward to the date of publication in every instance. Each time, too, it has been enlarged as new facts have been developed, or old ones utilised, until now it is about as near perfect as such a work could be.

The author is such a practical clinician, is so expert in the bedside use of therapeutic measures, and withal, is so competent a teacher, that he is enabled to write with an *ex cathedra* forcefulness vouchsafed to few. This lays an excellent foundation for the making of a text-book, and easily accounts for the popularity of this and kindred treatises from Dr. Hare's facile pen.

The arrangement of this book is beyond criticism, it is of easy reference, and contains just what the hurried searcher needs to help him in his every day work.

